# Vulnerability of migrant women during disasters: a scoping review of the literature

**DOI:** 10.1186/s12939-023-01951-1

**Published:** 2023-07-22

**Authors:** Monica Trentin, Elena Rubini, Awsan Bahattab, Mariarosa Loddo, Francesco Della Corte, Luca Ragazzoni, Martina Valente

**Affiliations:** 1 CRIMEDIM - Center for Research and Training in Disaster Medicine, Humanitarian Aid and Global Health, Università del Piemonte Orientale, 28100 Novara, Italy; 2 Independent Scholar, Vercelli, 13100 Italy

**Keywords:** Migrant women, Vulnerability, Intersectionality, Disasters, COVID-19

## Abstract

**Background:**

Disasters have an unequal impact on the population because of differences in conditions of vulnerability, exposure, and capacity. Migrants and women are among the groups that are at greater risk for and disproportionately affected by disasters. However, despite the large body of evidence that analyzes their vulnerability separately, disaster research that targets migrant women is scant. The aim of this scoping review was to analyze the published scientific literature concerning the vulnerability of migrant women and the consequent negative impact they experience during disasters.

**Methods:**

A literature search was conducted on December 15th, 2021 on Pubmed, Scopus, and Web of Science databases. No time filter was applied to the search. Information regarding the article’s main characteristics and design, migrant women and their migration experience, as well as about the type of disaster was collected. The factors responsible for the vulnerability of migrant women and the negative outcomes experienced during a disaster were extracted and inductively clustered in main themes reflecting several vulnerability pathways. The review followed the Joanna Briggs Institute methodology for scoping reviews and relied on the Preferred Reporting Items for Systematic Reviews and Meta-Analyses extension for Scoping Reviews (PRISMA-ScR).

**Results:**

After full text review, 14 articles met the inclusion criteria. All of them adopted a qualitative methodology and focused on COVID-19. The pandemic negatively affected migrant women, by triggering numerous drivers that increased their level of exposure and vulnerability. Overall, six vulnerability factors have been identified: legal status, poverty conditions, pre-existing health conditions, limited agency, gender inequality and language and cultural barriers. These resulted in nine impacts: worsening of mental health status, poor access to care, worsening of physical health conditions, fraud, exacerbation of poverty, gender-based violence, jeopardization of educational path, and unfulfillment of their religious needs.

**Conclusions:**

This review provided an analysis of the vulnerability factors of migrant women and the pathways leading to negative outcomes during a disaster. Overall, the COVID-19 pandemic demonstrated that health equity is a goal that is still far to reach. The post-pandemic era should constitute the momentum for thoroughly addressing the social determinants of health that systematically marginalize the most vulnerable groups.

**Supplementary Information:**

The online version contains supplementary material available at 10.1186/s12939-023-01951-1.

## Introduction

Disasters have an unequal impact on the population. Some groups, such as the elderly, migrants, women, disabled, or unhoused people suffer worse consequences than others because of differences in conditions of vulnerability, exposure, and capacity [1, 2]. The Health Emergency and Disaster Risk Management (H-EDRM) framework, developed in 2019 by the World Health Organization (WHO) to consolidate current approaches and practices aimed at reducing health risks and consequences of emergencies and disasters, identifies migrants and women as two separate groups that are at greater risk of and disproportionately affected by disasters compared to the general population [3].

In the context of disasters, *vulnerability* refers to the conditions determined by physical, social, economic, and environmental factors or processes which increase the susceptibility of an individual, a community, assets, or systems to the impacts of hazards. Vulnerability lies on the same spectrum of resilience as it often results in a diminished capacity that a community has to anticipate, cope with, resist, and recover from the impact of disasters. Vulnerable groups are more likely to experience disproportionate impacts and suffering in terms of mortality, morbidity, and losses when compared to the general population [4–12].

In the context of the COVID-19 pandemic, a recent global disaster that significantly impacted societies across the world [13], refugees and migrants were at increased risk of contracting the virus when living in overcrowded houses without access to basic sanitation [14]. Their typically compromised ability to access the health system due to financial, administrative, legal, and language and cultural barriers [15] was further undermined.

For women, vulnerability arises from the systematic differences in power relations and social hierarchies [16], as well as gender roles, which influence their socioeconomic status and level of agency [17–19]. During the COVID-19 pandemic, women experienced worse socioeconomic impacts, faced access constraints to sexual and reproductive care [20], and bore the burden of the increased responsibilities for childcare [21]. The positive correlation between gender-based violence (GBV) and disasters [22] was confirmed [20].

By adopting an intersectional lens that combines the vulnerabilities resulting from being a migrant or a refugee with those arising from being a woman, we can assume that disaster outcomes for migrant women are even worse than those affecting migrants and women if considered in isolation [23], especially in terms of socioeconomic and labor-related impacts as well as the impact related to the disruption of gender-based care [24]. Yet, despite the large body of evidence that analyzes the vulnerability of migrants and women in the context of a disaster, research that targets migrant women is scant. If several literature reviews targeting migrants and women as separate groups have been published [25–35], this is the first scoping literature review addressing migrant women’s vulnerability during disasters.

The increase in the frequency and intensity of disasters in the last years [36] with the projections for the near future [37] necessitate the prioritization of the most vulnerable groups. Understanding the impact of disasters on migrant women is essential to enhance their level of disaster preparedness as well as to improve their coping strategies.

The aim of this scoping review is to retrieve and analyze the published scientific literature concerning the vulnerability of migrant women and the consequent negative impacts they experience during disasters in their host countries. Specifically, the research was guided by the question: “How migrant women experience vulnerability during disasters in their host countries?”. Giving an answer to this question will provide an overview of migrant women’s vulnerability and will contribute to proper conceptual understanding of vulnerability – often ambiguously defined in the literature [38] – thus enriching the body of scientific research on this topic.

## Methods

The methodology for this scoping review was based on the Joanna Briggs Institute methodology for scoping reviews [39] and relied on the Preferred Reporting Items for Systematic Reviews and Meta-Analyses extension for Scoping Reviews (PRISMA-ScR) checklist [40].

For the purposes of this study, a disaster is defined as “a serious disruption of the functioning of a community or a society at any scale due to hazardous events interacting with conditions of exposure, vulnerability and capacity, leading to one or more of the following: human, material, economic and environmental losses and impacts” [1]. For operational purposes, the term “migrant” is used as an “umbrella term” to refer to women of any age who move away from their place of usual residence across an international border, temporarily or permanently, for a variety of reasons such as war, work, or family issues [41]. To account for the peculiarities of different migratory experiences, the definition of migrant as reported by the authors of the original articles has always been specified. This review focused on women that were migrants before the disaster occurred and not as a consequence of it.

### Search strategy

A literature search was systematically conducted on December 15th, 2021 on PubMed, Scopus, and Web of Science databases. The search strings (see Additional file 1) combined three different sets of terms, namely migrant-related, woman-related, and disaster-related ones. No restrictions or filters were applied to the research. The search covered all years from the origin of the databases until December 15th, 2021. After removal of duplicates, titles and abstracts of the remaining articles were manually screened by one investigator (MT) and those not complying with the inclusion criteria were excluded. All the full-text articles eligible for inclusion were reviewed independently by two reviewers (MT, ER) and discrepancies were resolved after discussion with the whole group. When articles’ full text was not available, the corresponding authors of the studies were contacted (n = 2). The references of the selected articles were also screened to identify any other relevant study to be included.

### Eligibility criteria

The study selection process relied on the following inclusion criteria: (a) the study deals with the vulnerability of migrant women during a disaster as reported by migrant women or by providers who served them during the disaster; (b) the study is an original one, using either qualitative or quantitative methodology. Exclusion criteria were: (a) the study deals with the vulnerability of migrants, without a specific focus on women; (b) the study deals with the vulnerability of women, without a specific focus on migrants; (c) the study considers female gender as one of many variables associated with mortality/morbidity without exploring the reasons for migrant women’s vulnerability; (d) the study is not an original one (e.g. review, letter to the editor). Studies dealing with HIV were not included in this review as per the definition of disaster adopted in this study. No studies were excluded because written in languages unknown by the authors; if potentially relevant articles had been found in languages unknown by the authors, external collaborators would have been contacted.

### Data extraction and analysis

A Google sheet was developed to extract relevant information from the included studies (see Additional file 2). The collected data included information on the article’s main characteristics and the study design, information about migrant women and their migration experience, information about the type of disaster that was experienced, migrant women’s vulnerability factors and the negative outcomes resulting from the disaster. A qualitative synthesis of study findings was supported by a phenomenological approach [42, 43] and an inductive analysis [44] focusing on experiences of migrant women during the disaster. Main themes reflecting several vulnerability pathways for migrant women during disasters have been identified.

## Results

**Fig. 1:**
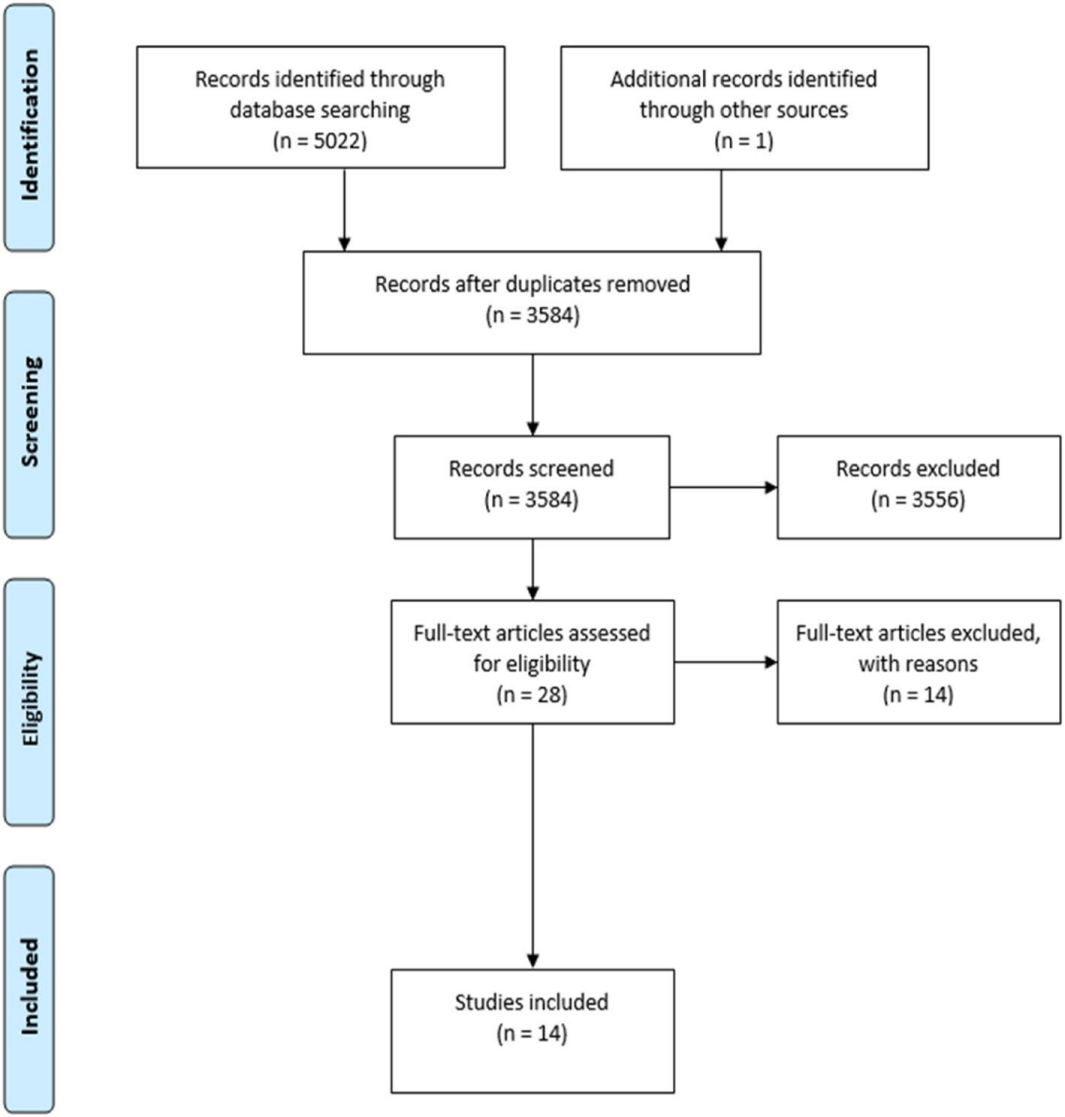
Study selection process

The search returned a total of 5023 articles. After removing duplicates, 3584 articles were eligible for abstract review, and 3467 articles were excluded because they did not deal with migrant women’s vulnerability during a disaster, while 90 articles were excluded because they were not original studies. One article was identified through manual search. In total, 28 articles met criteria for full-text review, and 14 articles met the inclusion criteria after full-text review. One study [45] was classified as a “viewpoint” by the journal, but it was included in the final list because the authors collected original data. Another study [46] collected data both from male and female participants, but it was included given the high relevance attributed to the latter. The study conducted by Marabello [47] included both male and female participants, but only information about women was reported in the present review. Detailed information regarding the selection of sources of evidence can be found in the PRISMA diagram (Fig. 1), while a comprehensive overview of the main characteristics of the studies is presented in Table 1.

**Table 1:** Characteristics of the included studies

**Author and year**	**Country**	**Study period**	**Study type and methodology**	**Objective**	**Population**	**Key findings**
Açıkalın et al. (2021) [48].	Türkiye	March 2020—June 2020.	Qualitative; semi-structured interviews.	To determine how the COVID-19 pandemic influenced integration of women refugees into Turkish society.	50 refugees.	The COVID-19 outbreak affected refugee women’s integration in an unprecedented way, especially with regard to education, economy, and social and family life. Their access to healthcare was also compromised. Refugee women stated that the pandemic positively influenced the security dimension of integration.
Angulo-Giraldo et al. (2021) [49].	Peru.	March 19—April 30, 2020.	Qualitative; Retrospective correlational study with an online survey.	To identify the impact generated by the media on Venezuelan migrant women in the context of the COVID-19 pandemic and to know how it relates to their emotional state and to compliance with health recommendations.	385 migrant women from 2 associations of Venezuelan migrants residing in Lima.	There is an association between media and emotional impact; none of the analyzed media provided reassuring information but disturbed the audience. In the context of the COVID-19 pandemic, the media sharpened migrant women’s feelings of concern, fear, terror, and anger.
Golesorkhi et al. (2020) [45].	United States of America (USA).	May—July 2020.	Qualitative; interviews and community observation.	To highlight the significance of community efforts in providing gender-responsive measures that address the specific needs and challenges of refugee women.	15 refugee women and representatives of organizations working with refugees.	Refugee women’s livelihoods have been impacted by the COVID-19 pandemic in several ways. These included job loss and barriers to access to healthcare, becoming essential workers and assuming additional caretaking roles, and finding themselves again in situations of limited mobility and social isolation.
Karajerjian (2021) [50].	Lebanon.	2021.	Qualitative; FGDs and semi-structured interviews.	To understand how refugee women who escaped the Syrian war cope with their exile and how it affects their mental health and well-being.	> 50 migrant women who have been visiting primary healthcare centers in Beirut and 2 social workers.	Syrian refugee women in Beirut had to face several hardships, such as paying rent, finding a job, accessing aid, and continuing to perform their caring roles. The COVID-19 pandemic has multiplied their personal and economic challenges, affecting participants’ mental health and overall well-being.
Lightman (2021) [51].	Canada.	January 1—March 30, 2021.	Qualitative; in-depth interviews.	To uncover the lived experiences of immigrant women healthcare aides (HCAs) working in Calgary's long-term care (LTC) sector during the COVID-19 pandemic.	25 immigrant women working as HCAs in LTC in Calgary.	The COVID-19 pandemic impacted the working lives of immigrant women employed in LTC facilities on a daily basis. Their experiences of economic and social exclusion have been exacerbated by the pandemic.
Lusambili et al. (2020) [52].	Kenya.	October 2020.	Qualitative; in-depth interviews.	To improve understanding of the impact of COVID-19 on women refugees' access to and utilization of antenatal, delivery and postnatal care.	15 patients from antenatal and postnatal care who are migrant women and 10 healthcare workers (HCWs) and community health volunteers.	Within the first 8 months of COVID-19, refugee women preference for home deliveries increased and health care workers reported having observed reduced utilization of services and delayed care-seeking. Fear, economic challenges, and lack of migrant-inclusive health system policies were key factors influencing home deliveries and delayed and low uptake of facility-based care.
Marabello et al. (2020) [47].	Italy.	Not indicated.	Qualitative; ethnographic study.	To investigate notions of visibility/invisibility of migrants in Italy and how they have been impacted by the COVID-19 outbreak and also related to the virus and its perception.	Diseased adult men, sex-trafficked women, and mothers of young children (0–5 years).	The COVID-19 pandemic had several repercussions on migrants and refugees living in reception structures in Bologna, and interrupted their personal trajectories and projects. After the implementation of policies that rendered invisible the condition of migrants in the months prior to the pandemic, migrants returned to a position of visibility as essential service workers.
Melov et al. (2021) [53].	Australia.	November 18—December 16, 2020.	Qualitative; semi-structured interviews.	To explore the experience of maternity clinicians serving a high migrant population during the COVID-19 pandemic.	14 maternity care clinicians working in a tertiary referral hospital in Sydney.	COVID-19 related travel restrictions resulted in loss of valued family support for migrant women’s families. As a consequence, male partners had to replace the role of absent overseas relatives.
Mutambara et al. (2021) [54].	South Africa.	July—October 2020.	Qualitative; interviews.	To illustrate the ways in which COVID-19 has exacerbated refugee women's insecurity and intensified structural violence which renders them vulnerable.	26 refugee women.	The COVID-19 pandemic, together with mitigation measures, has impacted refugee women well-being, exacerbated their insecurities, and intensified structural violence. If left unaddressed by government, NGOs and civil society organizations, the impacts of the pandemic could lead to long-term violence and insecurities.
Nardon et al. (2021) [55].	Canada.	August 2020.	Inductive, qualitative and elaborative study; online questionnaire.	To explore how the COVID-19 pandemic impacted skilled newcomer migrant women’s labor market outcomes and work experiences.	69 migrants; exact legal status not specified.	The COVID-19 pandemic pushed skilled immigrant women towards unemployment, lower -skilled or less stable employment. Most study participants had their career trajectory delayed, interrupted, or reversed due to layoffs, decreased job opportunities, and increased domestic burden. The gendered nature of the pandemic and the reliance on work-from-home arrangements and online job search have increased immigrant women’s challenges due to limited social support and increased family responsibilities.
Phillimore et al. (2021) [46].	United Kingdom (UK), Türkiye, Tunisia, Sweden, Australia.	April 14—April 28, 2020.	Qualitative; interviews.	To examine the condition of forced migrant survivors of sexual and gender-based violence (SGBV).	52 forced migrants that were also SGBV survivors (48 female, 4 male) and service providers (e. g. SGBV personnel, psychologists and social workers).	The conditions generated by the COVID-19 pandemic have added an additional layer of disadvantage for forced migrant women survivors of SGBV. The experiences of forced migrants have been shaped by multiple intersecting inequalities: those with irregular immigration status, without access to public funds, and with caring responsibilities experienced worse outcomes.
Sabri et al. (2020) [56].	USA.	Not indicated.	Qualitative; interviews.	To understand immigrant survivors and service providers’ perspectives on the impact of COVID-19 on survivors’ health and safety, the quality of services and suggestions on how to mitigate the risks for increased intimate partner violence (IPV).	45 immigrant women. 17 key informants/ service providers with experience in serving survivors of IPV.	All participants described a reciprocal and reinforcing relationship between increased life stressors and IPV due to the COVID-19 pandemic and containment measures put in place. Support strategies have also been suggested from the participants.
Mingo (2021) [57].	Cuba.	Not indicated.	Analysis of a single interview whose content has been systematized.	To examine from the perspective of race, gender, and class the consequences of the COVID-19 pandemic on the participant’s personal and work life as well as in her relationship with the host country.	1 migrant woman.	The COVID-19 pandemic caused uncertainty and pessimism. The woman mentioned in the article experienced episodes of discrimination due to the intersectionality of race, gender, and class.
Simic (2021) [58].	Australia.	Not indicated.	Personal essay.	To offer a personal reflection on life in Australia during the COVID-19 pandemic, in particular on what it means for a migrant woman with a complex, traumatic past to be forcibly separated from her family during lockdown.	1 migrant woman.	The COVID-19 pandemic triggered personal struggles such as forced separation and causing trauma from the past to come to light again.

### Characteristics of the studies

All the studies included focus on the COVID-19 pandemic and therefore were conducted and published between 2020 and 2021.

All the 14 studies were qualitative. The most used methodology was interviews: in some cases, details about the type of interviews, namely in-depth [51, 52] or semi-structured [48, 50, 53], were included. Two studies used an ethnographic approach [45, 47], and in one study focus group discussions (FGDs) were also conducted [50]. In two studies a questionnaire was administered [49, 55] (Table 1).

The main focus of the studies, as reported by the authors, revolved around six main themes: the relationship between migrant women and the host community [45, 47, 48, 57], the exacerbation of GBV and insecurities during the pandemic [46, 54, 56], mental health issues as a consequence of COVID-19 or the containment measures [49, 50, 58], the impact of the pandemic on migrant women’s working life [51, 55, 57], maternal care [52, 53], and perceptions and attitudes towards COVID-19 [47].

In 13 out of 14 articles the study population was composed of migrant women. In one article [53], the impact of the pandemic on migrant women was explored exclusively through interviews with maternity care clinicians. In other five articles [45, 46, 50, 52, 56], professionals such as social or health workers, service providers, and community representatives were involved. In two articles migrant men were also included [46, 47] (Table 1).

### Migrant women

An overview of demographic characteristics of migrant women is presented in Table 2. However, in some of the reviewed studies, the demographic information was vaguely described or not reported. The type of migrant, as reported by authors, was forced migrant [46], landed immigrant [51], refugee [45, 47, 48, 50, 52, 54, 55], skilled migrant [55, 58], applicant for refugee status [49], migrant carrying a passport with humanitarian visa [49], migrant with temporary permit to stay [49], and asylum seeker [47]. The legal status of migrant women was not specified in three studies [53, 56, 57] and another study only reported their ethnicity [56].

**Table 2:** Demographic characteristics of migrant women

**Author and year**	**Type of migrant**	**Home country**	**Host country**	**Length of stay in the host country**	**Type of disaster experienced in the host country**
Açıkalın et al. (2021) [48].	Refugees.	Afghanistan (3), Iraq (6), Somalia (7), Syria (34).	Türkiye.	Not indicated.	COVID-19 pandemic.
Angulo-Giraldo et al. (2021) [49].	Reported only for some participants: applicants for refugee status; migrants carrying a passport with humanitarian visa; with a temporary permit to stay.	Venezuela.	Peru.	Not indicated.	COVID-19 pandemic.
Golesorkhi et al. (2020) [45].	Refugees.	Not indicated.	USA.	Since 2011.	COVID-19 pandemic.
Karajerjian (2021) [50].	Refugees.	Different regions of Syria, primarily Deir Ez-Zor, Aleppo, Reef Aleppo, Idlib, Ar-Raqqa.	Lebanon.	Reported for 3 women: from 2013 (2 years), from 2015 (1 year).	COVID-19 pandemic.
Lightman (2021) [51].	Landed immigrants.	Bosnia (1), Democratic Republic of Congo (1), Eritrea (2), India (7), Nigeria (3), Philippines (9), Romania (1), Sudan (1).	Canada.	Not indicated.	COVID-19 pandemic.
Lusambili et al. (2020) [52].	Refugees.	Antenatal: Somalia (6), Tanzania (2), Uganda (1), Eritrea (1); Postnatal: Somalia.	Kenya.	Not indicated.	COVID-19 pandemic.
Marabello et al. (2020) [47].	Refugees or asylum seekers.	Not indicated.	Italy.	Information reported only for a woman (1 year).	COVID-19 pandemic.
Melov et al. (2021) [53].	Not indicated.	Not indicated.	Australia.	Not indicated.	COVID-19 pandemic.
Mutambara et al. (2021) [54].	Refugees.	Democratic Republic of Congo (16), Burundi (7), Rwanda (3).	South Africa.	Not indicated.	COVID-19 pandemic.
Nardon et al. (2021) [55].	Skilled migrants; refugees.	26 different countries, specified for 10 women: Algeria (1), Bangladesh (1), Brazil (3), China (1), Egypt (1), Iran (1), Nigeria (1), Yemen (1).	Canada.	Less than 5 years.	COVID-19 pandemic.
Phillimore et al. (2021) [46].	Forced migrants.	Albania, Camerun, Congo, Eritrea, Gambia, Ghana, Guinea, Iraq, Lebanon, Malawi, Namibia, Nigeria, Sierra Leone, Sudan, Syria, Türkiye.	UK, Türkiye, Tunisia, Sweden.	Not indicated.	COVID-19 pandemic.
Sabri et al. (2020) [56].	Not indicated.	The ethnicity, but not the exact country of origin, of some survivors is mentioned: Asian, African.	USA.	Not indicated.	COVID-19 pandemic.
Mingo (2021) [57].	Not indicated.	Cuba.	Spain.	10 years.	COVID-19 pandemic.
Simic (2021) [58].	Skilled migrant.	Former Yugoslavia (Bosnia).	Australia.	The author had been traveling for ten years but was not able to obtain any long - term visa before entering Australia. At the time of writing the article she had been living in Australia for 15 years and she has been holding an Australian passport for almost 8 years.	COVID-19 pandemic.

Age of migrant women was not indicated in six articles [45, 49, 51–53, 55], while, in other cases, it was reported in a non-standardized way, and did not include data about the entire group of participants.

**Fig. 2:**
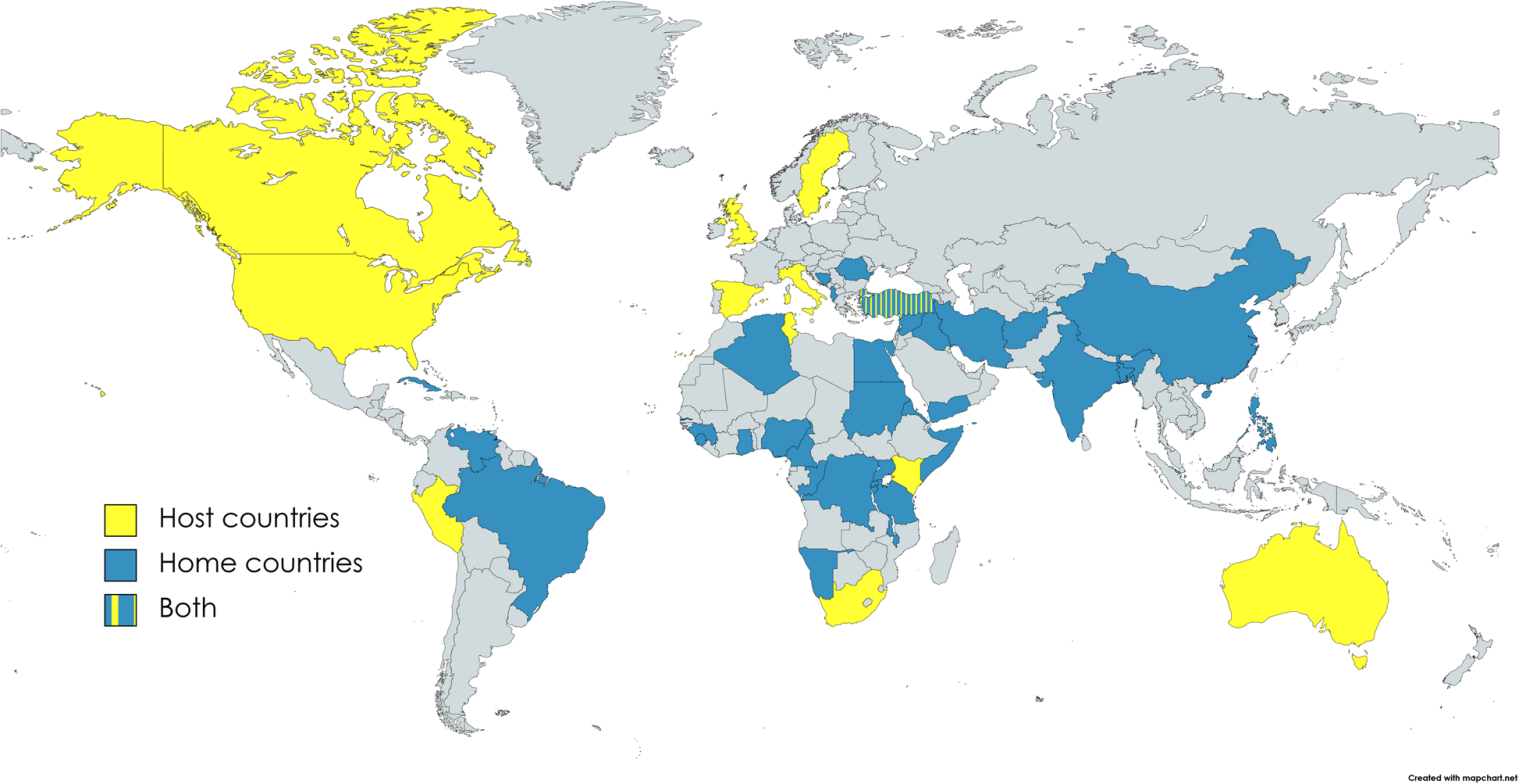
Host and home countries of migrant women. Note that while host countries were always reported by the authors of the studies, information on the home countries was missing in some articles. For more information see Table 2

The home and host country of migrants are presented in Fig. 2. It should be noticed that one study mentioned the country of origin of a limited number of participants [55] and, in another study [46] it was not clear if this information was provided for all participants.

The length of stay in the host country was mentioned in six studies ranging from one to 15 years [45, 47, 50, 55, 57, 58]. In eight cases, this data was not reported [46, 48, 49, 51–54, 56], while in other two cases the information was provided only for a limited number of participants [47, 50] or was vague [55].

### Vulnerability pathways

Migrant women were negatively affected by the COVID-19 pandemic. Together with the containment measures put in place to limit the spread of the virus, the pandemic triggered numerous drivers, which increased migrant women’s levels of exposure and vulnerability, thus resulting in greater impact. In this section, the pathways that generated from a condition of vulnerability and resulted in a negative impact for migrant women are reported.

**Fig. 3:**
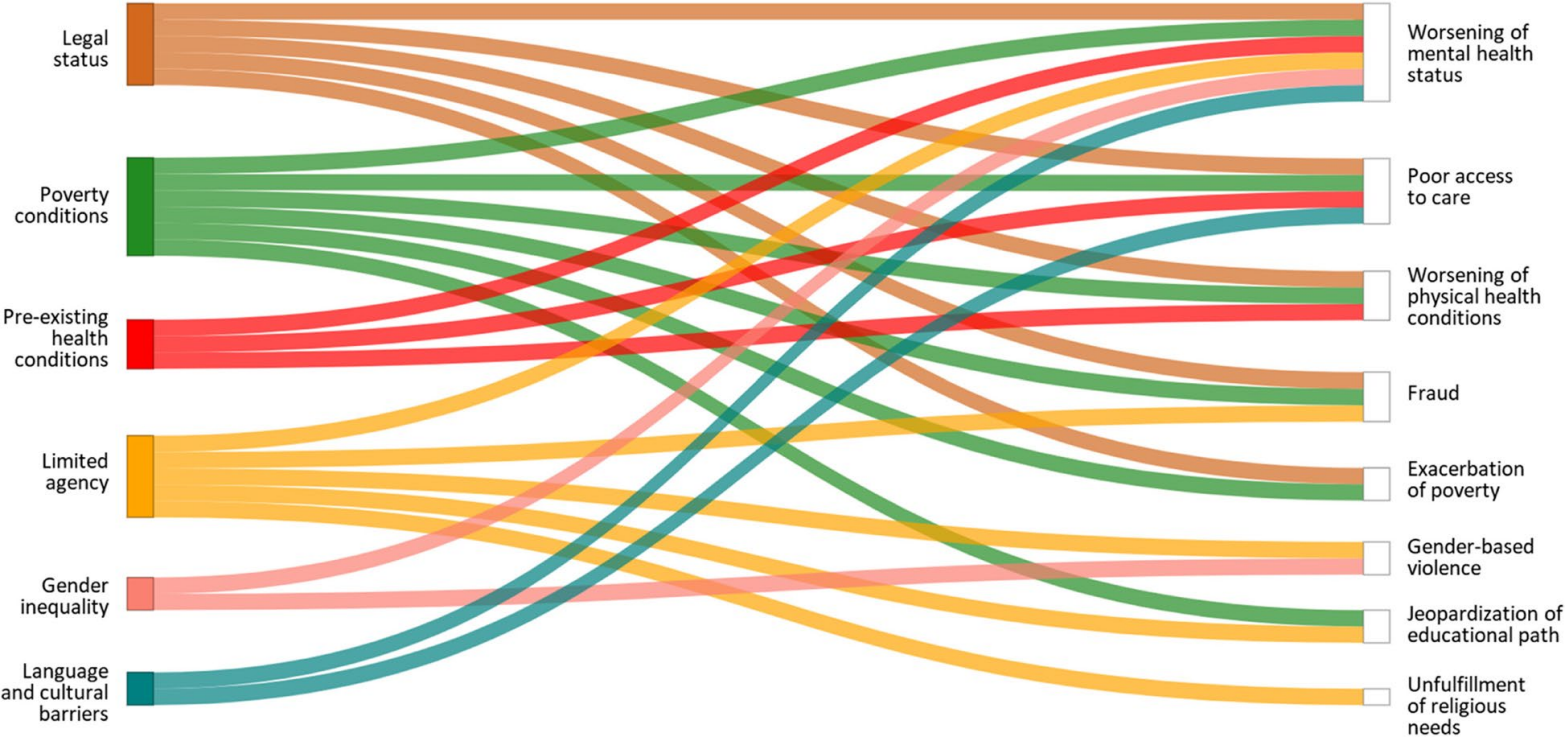
Flow diagram outlining migrant women’s vulnerability pathways during disasters

Overall, six vulnerability factors have been identified: legal status, poverty conditions, pre-existing health conditions, limited agency, gender inequality, and language and cultural barriers. These resulted in nine impacts: worsening of mental health status, poor access to care, worsening of physical health conditions, fraud, exacerbation of poverty, GBV, jeopardization of educational path, and unfulfillment of religious needs (Fig. 3). A graphical representation depicting the pathways in a more detailed way can be found in the Additional file 3.

### Legal status

One of the vulnerability factors that affects migrant women the most is their legal status, as it compromises their job opportunities, access to care, and living conditions [45–47, 50, 52, 54–57]. The study of Lusambili [52] reported that in Kenya government policies such as *Linda mama mtoto* (short for *Linda Afya ya mama na Mtoto*), which allows pregnant women and infants to access affordable maternity care, turned out to be discriminatory against refugees, in particular during COVID-19. Discriminatory policies, especially when combined with migrant women’s poverty conditions, were exasperated by the shortage of medical staff during the COVID-19 pandemic, the lockdown measures, and the fear of contracting the virus, which ultimately led to an increase in home deliveries and lower utilization of reproductive, maternal, newborn and child health services, posing serious risks to both mothers and their babies (e.g., vaccinations missed) [52].

Moderator: So, the migrants don’t qualify for the Linda mama mtoto?

Respondent [Community Health Volunteer]: They qualify, but the program through NHIF [National Health Insurance Fund] refunds for deliveries for hosts [Kenyans] only, but for the migrants there is no refund. So, the facility normally says that they are doing a useless job. That is what they say. Just last week, we had like four migrants from Uganda. They delivered while I was there present, but they were saying that they are doing a useless job. (Lusambili, p. 5) (Host country: Kenya).

Racist and discriminatory attitudes from healthcare professionals, the police and the general population towards migrant women were exasperated during the COVID-19 pandemic, thus negatively impacting their mental health status [45, 49–55, 57]. Lightman [51] reported how being a healthcare assistant was perceived as an “immigrant job” and, with the start of the pandemic, Asian healthcare assistants employed in long-term care (LTC) facilities in Canada were blamed by the residents for bringing in the virus. Discrimination was also impacted by employment competition, aggravated by COVID-19 pandemic [48, 49]. In the United States of America (USA) migrant women feared for their own safety due to the political climate related to immigrants and the increase in gun purchase for self-protection during the containment measures in the general population [56]. On a positive note, some of the participants in one study received financial support from their neighbors while others expressed positive feelings towards their host country, in relation to hygiene, safety, and social services [48].

The closure of government facilities during the lockdown caused delays in the issuance of new or renewed visa and residence permits, increasing migrant women’s sense of uncertainty [46, 47, 54, 56]. When undocumented, migrant women feared being deported [46, 56] and for this reason they experienced anxiety and distress when seeking medical help [46]. They were also worried they would not have been a priority if a ventilator was needed, and that mortality rates in the migrant population would not have been reported by the authorities, making them invisible [46].

During the pandemic, the job and income of migrant women was often compromised and because of their legal status and their holding a temporary visa, or due to the fact they were employed in informal jobs, they were unable to access state benefits and were excluded from the safety net implemented for the general population [46, 54–56]. The extreme marginalization in which migrant women found themselves made them more vulnerable when fraudulent organizations tried to lure them [54].

### Poverty conditions

Migrant women live in a condition of vulnerability because of poverty [46–48, 50–52, 54], and this was exacerbated during the COVID-19 pandemic. Due to their legal status, migrant women face precarious employment situations, are forced into informal jobs such as working as childminders, hair-dressers, street vendors, cleaners, car guards, domestic assistants [49, 54], or unfavorable contracts (e.g., the zero-hour contracts in the United Kingdom (UK)) [46]. The difficulty in getting a permanent or full-time job was reported. Consequently, to get to the end of the month migrant women had to work at more than one job site or extra shifts [51]. In other cases, they worked illegally and without any protection and safeguards [46], or did not work at all and had to depend either on other household members (e.g., the husband) or on welfare or charity (e.g., Non Governmental Organizations (NGOs)) [46, 48]. One study reported that migrant women received help from churches, however this didn’t resolve their financial difficulties [54]. This situation was exacerbated by migrant women having children and sometimes being single parents [46, 54, 55]. Due to the containment measures that were implemented by government states, the COVID-19 pandemic negatively impacted migrant women’s working life and economic situations, often exacerbating their pre-existing precarious socio-economic status [46, 48, 50, 51, 54–56], also because their working conditions prevented them from saving money [46, 54]. High numbers of job lay-offs or downturning were reported, particularly among informal workers [50, 54–56].

I was working at this factory where we were packing plastics. For us it was already difficult because we had gone for 3 months without working and we were not being paid and when they said lockdown that was it, they never paid us. (Mutambara, p. 708) (Home country: Rwanda; host country: South Africa)

For people like us who are self-employed and into buying and selling goods it was difficult because we could no longer sell our products because everyone was now locked in their houses. (Mutambara, p. 707-708). (Home country: Burundi; host country: South Africa)

The single site working policy introduced by the Alberta government in Canada in April 2020 as a strategy to limit cross-contamination of the virus across multiple LTC facilities significantly impacted migrant women’s finances since they had been working at more than one job site to get through the month [51]. The study of Nardon involving skilled migrant women, reports that they suffered a delayed start of their career, a reverse career trajectory, or its interruption [55]. Because of their migrant status or informal job, migrant women were often ineligible for labor welfare and emergency government support, as opposed to the rest of the population [45, 46, 51, 55].

I don’t have any benefits. I cannot go to the dentist. I cannot go to a massage. I cannot go to take my daughter to psychology, psychiatry, whatever, because she has some anxiety problem since this Covid started. I cannot take her because it costs money. (Lightman, p. 6) (Home country: Romania; host country: Canada)

Due to the economic crisis that was experienced, migrant women suffered food shortage, hunger, and dehumanization [46, 54, 56]. They were unable to pay the rent and sometimes were evicted or were threatened to be evicted [46, 50, 51, 54, 56]. All these elements seriously impacted migrant women’s mental health by causing them distress and anxiety.

[The landlord] wanted his money and he came, and he cut our water and electricity, so we were really suffering. I was sleeping in the dark with my child. (Mutambara, p. 710) (Home country: Burundi; host country: South Africa)

When unprovided with bank accounts, migrant women were excluded from online bill payments or using public transport, which during the pandemic shifted to only accepting card payments. This exacerbated their immobility and hindered their possibility to rely on cheaper resources [46].

Many of the migrant women are also affected by digital poverty, with no or limited access to internet, television, and phones [45, 46, 48]. Due to the containment measures, learning opportunities, support groups, and meetings with social workers transitioned online and migrant women were often excluded with impacts on their mental well-being and jeopardization of their educational path [46, 48, 50].

Because they were seriously in need of help, migrant women were induced to trust unnamed NGOs that took advantage of the most vulnerable by fraudulently claiming COVID-19 relief measures.

There is this Non-Profit organization, I don’t recall their name. I contacted them seeking help and they took my details and whatnot, my asylum and all and only for me to realize that they never helped me with anything. And when I tried to WhatsApp them, I realized that they had blocked me on their number, and I went and looked for their social media page and they had also blocked me. (Mutambara, p. 717) (Home country: Democratic Republic of the Congo; host country: South Africa)

Migrant women are often forced to live in inadequate spaces due to their poverty and legal status. Many participants of the study conducted by Phillimore [40] in the UK, Tunisia, and Türkiye were living in shelters, shared accommodations, and overcrowded housing with shared kitchens and toilets. One Syrian woman living in Beirut compared her house to a prison, since it was very small and she lived there together with her four children [50]. This situation turned out to be particularly tough during the COVID-19 pandemic because the risk of contagion was much higher in overcrowded houses or migration reception systems. Because of the great amount of time spent at home during the lockdown, tensions among members of the family were also common [50, 54].

Poverty conditions prevented women from accessing healthcare. Firstly, migrant women feared unaffordable charges [46, 52]. The study of Lusambili describes the situation of women who were forced to deliver at home, since there was a lack of affordable hospitals offering maternity services and government maternity services were unavailable [52].

[Migrant women] do not have money and cannot afford to go to private hospitals as they are very expensive. … Some of the refugee women who were expectant would even deliver alongside the road because they did not have money. They went through difficulties. (Community health worker, Lusambili, p. 5) (Host country: Kenya)

Secondly, migrant women’s housings are often in peripheral areas, with hospitals far to reach. With reference to the study of Lusambili, the distance from hospitals is described as one of the reasons that often force migrant women to home birth [52]. Beyond all the risks it entails, delivering at home may result in difficulties in getting a child’s birth certificate, forcing families to pay in order to receive it, thus aggravating even more their already precarious situation.

Respondent: Yes, I got some mothers post-natally [post natal] that wanted me to guide them on how to get birth certificates. You know when you deliver at home it gets hard to get a notification. I got quite a number whom I had to guide on the process of getting birth certificates. They had to report to the chief.

Moderator: That is a long process.

Respondent: You know a sub-chief had to be paid. Everything is money here in Kenya.

(Community health worker, Lusambili, p. 4) (Host country: Kenya)

### Pre-existing health conditions

Migrant women’s pre-existing poor health conditions were reported in six studies [47–50, 52, 58]. In two articles [47, 52] some of the women were said to be HIV positive. In other cases, they suffered from kidney failure, hypertension, allergies, or diabetes [50, 53, 54]. In other instances, their health-related vulnerability factor was pregnancy [52, 53].

During the pandemic, fear of COVID-19 prevented migrant women with chronic health issues from accessing healthcare facilities, causing them to stop attending their check-ups and inhibiting them from receiving the necessary treatments [54].

It brings fear, this Corona affected us with fear, there is a fear to even go and get medication for blood pressure at the hospital because I will be thinking that maybe if I go there and get into contact with one person who has Corona then I will die. (Mutambara, p. 714) (Home country: Burundi; host country: South Africa)

The priority given to patients affected by COVID-19 at the expense of non-COVID-19 ones resulted in the failure to receive care for chronic conditions [52, 54]. For example, the rearrangement of hospital activities implemented in order to cope with the health emergency resulted in the disruption of antenatal classes [53]. The study of Lusambili reported that viral load taking for HIV positive mothers was postponed and that because of the curfew women in labor could not access the hospital after 7 p.m., meaning that they had to deliver at home [52]. Açıkalın reports testimonies from migrant women who could access care only to get their children vaccinated and to get cancer treatment [48]. The fact that migrant women were sent away from hospitals because they were not considered a priority, sometimes resulting from and into medical xenophobia, ultimately led them to the use of do-it-yourself remedies [54].

I had a very sharp stomach pain and I went to the clinic but the way the nurses received me. They said they cannot help me because they only have to treat people for Coronavirus. They never attended to me and I was just sitting there in deep pain and then had to go back home. Since then, I have been sick, I was having flu. I never went back to the hospital and then I used lemon, ginger and garlic, that is what I used. I never went back to the hospital because I was scared of how the nurses would treat me because the last time, they were even rude to me. (Mutambara, p. 714-715) (Home country: Burundi; host country: South Africa).

Testimonies of migrant women who portrayed their experience of accessing healthcare services in a positive way were also found [48].

Another sphere of vulnerability is connected with migrant women’s mental health, as reported in four articles [47, 49, 50, 58]. For example, Syrian women who have been living in Lebanon after fleeing war suffer from anxiety and are constantly stressed and scared, having nightmares, difficulty in performing regular activities and experiencing hair loss [50]. In addition, in the Arab region mental health is considered a taboo issue and therefore migrant women might be discouraged from seeking help [50]. Stigma around this topic was also mentioned in Sabri [56]. The COVID-19 pandemic and the lockdowns triggered memories of migrant women’s traumatizing events [45–48, 50, 53, 55, 57, 58], hence increasing risk of self-harm and suicidal thoughts [46]. In the study of Marabello, when social workers proposed to a woman to visit a psychologist, she reacted with distress, refusing that and claiming her condition was a result of witchcraft [47].

The lockdowns intensified migrant women’s social isolation and loneliness resulting in the worsening of their mental well-being [45, 47, 48, 50, 52, 53, 55, 57, 58]. The situation was particularly hard also for those women that moved to a new country just before the COVID-19 pandemic, or those who had not yet developed their social capital. This had repercussions on migrant women’s psychological adjustment, since they were frustrated about the integration process and had the feeling of being stuck and not creating new opportunities for the future [55]. The positive experience of receiving support by other refugees’ families was described in one study [48].

According to the clinicians interviewed in the study of Melov, in Australia the lack of family support from overseas was one of the biggest challenges for pregnant and newly mothers migrant women, since in South Asia and China they are used to including their whole family in the childbearing process and in the postpartum period [53].

Due to the disruption of the social services determined by the containment measures, migrant women could not attend support groups and meet social workers or other professionals [46, 50]. On a positive note, one study reported that telehealth appointments with multilingual psychiatrists and sharing videos with advice on mental health were put in place [45].

### Limited agency

COVID-19 containment measures further limited women’s autonomy and decision-making capacities [47, 48, 52, 54, 58]. For example, in the study of Marabello a woman expressed her desire to leave her child with a friend of hers while she was not at home, but her request was denied by social workers because, following the government guidelines for limiting the spread of the virus, she had to rely only on flat mates living in the same reception system [47]. A woman living in Australia said that through the indefinite shutting of Australian borders the government “abruptly took away [her] agency and freedom of movement, so hard won, leaving [her] feeling trapped, helpless, and angry” [58] and this feeling was shared by some of the participants to Phillimore’s study [46].

The educational path of migrant women as well as their religious needs suffered from the lockdowns [46, 48] and provoked a reduction or a nullification of their decision-making capacities. On the one hand women had to delay or interrupt their educational efforts, which may have helped them improving their integration within their host communities, for example learning a new language, or developing skills helpful in finding a job. On the other hand, their decision to practice a religion together with other devotees was obstructed, limiting the way they could express their freedom of faith and their belonging to a community [48].

The lockdown and the closures of support programs for GBV survivors [46, 50] represented another obstacle in their path towards empowerment and the reestablishment of their decision-making capacities. In particular, the inability to leave the relationship for some of the survivors was connected with pre-existing vulnerability factors, such as their legal status and poverty conditions, and with drivers such as the disruption of social services, which hindered them also from reporting abuses [56].

Moreover, the pre-existing limited agency prepared the ground for the victimization provoked by fraudulent initiatives of NGOs who pretended to try and help the women [54]. The limited agency endured by migrant women, aggravated by the disruption of social services, negatively influenced their mental well-being [48, 58].

### Gender inequality

During the COVID-19 pandemic school closures and the lack of non-parental childcare options together with remote work challenges have exacerbated the disproportionate gendered division of housework and childcare in the home [48, 55, 56], increasing even more the household burden of migrant women. As a consequence, their mental health conditions worsened due to stress and anxiety [46].

Another negative outcome for migrant women found in the included literature was the increase in frequency and intensity of occurrences of GBV, in particular sexual and intimate partner violence (IPV), as a consequence of the enforcement of stay-at-home orders [46, 48, 50, 54, 56]. These amplified stalking and monitoring behaviors, as well as other forms of control, namely financial, trying to get survivors pregnant or threats to infect them with COVID-19 [56].

The man is just drinking beer all the time. If you ask where did you get money to buy beer when we do not even have money for food, it will be trouble, they just want to drink beer and the other thing is that because they are not working and they are having pressure, all the pressure they direct at us – the women. So, we as women, we are suffering. (Mutambara, p. 712) (Home country: Burundi; host country: South Africa)

If you are in the same house with an abusive partner, anything could be a trigger. If you can’t get out, then don’t get into any compromising situation. Try to stay quiet, to be as nice as you can. (Sabri, p. 1304) (Host country: United States)

Sometimes migrant women were concerned for the well-being of their children, who were also subjected to violence [46, 54, 56]. One study described how community babysitting, that is members of the woman’s social circle volunteering to take care of her children while at the same time protecting her from the abuser, was dismantled during the COVID-19 pandemic due to the containment measures [56].

The economic crisis provoked by the pandemic heavily aggravated migrant women’s dependency on their perpetrators, preventing them from leaving a threatening domestic environment [46, 54, 56]. In other instances they were sexually and financially exploited by their partners, as reported in one study describing girls being forced into prostitution [46].

Migrant women who experienced GBV could not attend support groups, meetings with social workers or other professionals and empowerment programs in person [46, 50] and when these activities moved online, they were often excluded because of the lack of access to the internet [46, 50]. The probability of being caught in the act when using virtual support services, and the associated risk for increased violence, was also reported [50, 56]. This situation further aggravated their poor mental health conditions, exacerbated by the fact that some of the women were left to fend for themselves [54, 56].

On a positive note, one study mentioned strategies used to help survivors of GBV, from providing technology, financial assistance, referral to safe shelters and mental health services to suggesting safe ways to cope with the abuser or to signal abuses [56]. Another study reported more apparent gender equality in providing support during pregnancy, parenting and division of household tasks within migrant couples during the pandemic [53].

### Language and cultural barriers

Language and cultural barriers could prevent migrant women from accessing services as well as from integrating in their host community [45, 46, 48, 53]. For example, Açıkalın explains how in Türkiye this issue represents an obstacle to migrant women’s interaction with the Turkish people, in finding a job and in accessing services available to them, including healthcare, and how this was further exacerbated in the context of the COVID-19 pandemic, with possible repercussions on their psychological well-being [48].

During the pandemic, because of language barriers, migrant women faced challenges in acquiring information about COVID-19 – from how to protect themselves to how to access services and resources – both on the government websites and in the media. Women had instead to rely on information provided by NGOs who made it available in different languages [45, 46]. However, when this service was present, it required time and, since the guidelines changed quickly, this made it impossible for migrant women to keep up with them through translation [45].

## Discussion

This scoping review explored the vulnerability factors of migrant women and the negative impacts they experienced during a disaster. Across the 14 studies included, legal status [45–47, 50, 52, 54–57] and poverty conditions [46–48, 50–52, 54] were the vulnerability factors related with most of the reported negative impacts, followed by limited agency [47, 48, 52, 54, 58], having pre-existing physical and mental health conditions [47–50, 52, 58], gender inequality [46, 48, 50, 54–56], and language and cultural barriers [45, 46, 48, 53]. As for the negative impacts experienced by migrant women, the worsening of their mental health status was the one influenced by all the identified vulnerability factors [45–51, 53–58], followed by poor access to care [45, 46, 48, 52–54], worsening of physical health conditions [48, 52, 54], fraud [54], exacerbation of poverty [46, 50, 51, 54–56], GBV [46, 48, 50, 54–56], the jeopardization of their educational path [46, 48] and unfulfillment of religious needs [48]. Although these pathways are presented in this review as compartmentalized, vulnerability factors and negative outcomes are deeply interconnected, constantly influencing and reinforcing each other. In addition, some negative impacts are provoked by the same vulnerability factor, which however follows different vulnerability pathways.

All the included studies dealt with the COVID-19 pandemic. Therefore, we can assume that the research interest in migrant women’s vulnerability during disasters is recent and emerged during the pandemic. Although some vulnerability factors such as poverty conditions and language and cultural barriers relate to all types of disasters, the lack of evidence from other contexts prevents us from fully exploring the wide spectrum of negative impacts that migrant women may experience during these events and to generalize our findings.

In other instances, the methodology presented some flaws. In some studies, the number of participants [50], the modality of recruitment [48], and the number of interviews that were conducted was not reported [45, 50]. In the study of Karajerjian it is not clear if the information reported was obtained from the FGDs or from personal communication via WhatsApp messaging with migrant women [50]. According to the authors of this review, migrant women’s point of view and experiences were not given enough space in three studies [45, 47, 52].

Migrant women’s vulnerability as presented in this review should be understood by adopting an intersectional approach. Intersectionality has been developed by Kimberlé Crenshaw in order to provide a framework for understanding the interrelated oppressions of African-American women [59]. It is also a useful analytical tool to express how different factors contribute to the marginalization of specific groups of people and individuals [2]. In fact, people’s degree of vulnerability depends on the complex relationship between “different axes of inequality” [60] provoked by social structures and constructions [61]. In the present review, it is the combination of migrant women’s status as “migrants” and as “women” with the socially constructed gender roles that this entails that makes them more likely to experience a worse impact in case of disaster. This also amplifies the vulnerability migrant women experience in comparison to non-migrant women and to migrant men. A striking example of how an intersectional approach sheds light on migrant women’s vulnerabilities is the worsening of their mental health due to the pandemic. This was caused by the disproportionate gendered division of housework and childcare at home during the pandemic – activities typically attributed to women due to socially constructed gender roles – together with the uncertainty arising from the suspension of services related to their legal status, and the exacerbation of their socioeconomic precarity due to job losses or downturning.

The great impact that the COVID-19 pandemic had on migrant women’s mental health allows us to reflect on the consequences of suspending in-person social services without finding inclusive solutions. Moving social services online resulted in the exclusion of those without computer, telephone, or the internet, or of survivors that needed GBV-related support but could not talk because they were forced at home with their perpetrators. This shows the importance to tailor a disaster response plan to the local context and the needs of specific communities, considering not only the impact of the disaster event itself, but also the snowballing consequences of containment measures on the most fragile and vulnerable groups.

The COVID-19 pandemic negatively impacted migrant women’s agency [47, 48, 52, 54, 58]. On one hand, migrant women were deprived of opportunities to build their lives in a new country. On the other hand, the coping mechanisms through which they could have dealt with the crisis were also destabilized. For example, religious coping proves to be a way of expressing agency and to have positive effects on migrants’ mental well-being [62]. However, migrant women could not fulfill their religious duties due to the containment measures [48]. Migrant women’s economic and legal dependence on perpetrators of violence during the pandemic vitiated their decision-making process and denied them the possibility to escape from an unsafe environment together with their children.

This review reported an increase in GBV during the COVID-19 pandemic, confirming previous findings [35]. From the studies included in this review, it has emerged that GBV survivors could not seek help because the economic crisis increased their dependence on their perpetrators [46, 54] and because social support was not always available. A study recently conducted in Italy – published out of the search timeframe of this review – reported how inclusive language and cultural mediation services were lacking and this prevented migrant women from seeking support; awareness campaigns were launched only in Italian and some social workers did not speak a language known by migrant women [63].

Although health promotion should be inclusive, multilingual, and multicultural [64], language barriers constituted a great obstacle in accessing information about the COVID-19 pandemic in terms of mode of transmission of the virus, use of personal protective equipment (PPE), services available, and bureaucratic information [45, 46, 48, 53]. Moreover, the notions of “danger” and “disease” vary across cultures as well as the meaning attributed to PPE such as masks [65]. In the study of Marabello, for example, a Nigerian migrant woman experiencing distress and anxiety attributed these symptoms to witchcraft instead of associating them with the abrupt interruption of her projects caused by the restrictions and the re-emergence of traumatic memories. Her quote “I already told you that the invisible can kill you, and now White people will finally understand” ([41], p.254) exemplifies her feelings towards the White/Black relations, in particular the feeling of invisibility of the causes and the sufferings of people from the Black community provoked by White people’s indifference and downplay. It is evident how cultural mediation is crucial to avoid marginalization of migrant women during disasters, and this needs to be included in countries’ disaster risk management and emergency planning [63].

It clearly emerges that migrant women were more negatively affected by the COVID-19 containment measures, such as the lockdowns, the social distancing and the travel restrictions, than by the pandemic itself. In this regard, some scholars pointed out how these measures constitute a “privilege”, not suiting certain communities [66, 67, 68]. Remote work, for example, was not possible for those who worked in the informal sector, as in the case of some members in the target group of this study. At the same time, social distancing was not an option for those who were living in small and overcrowded spaces or within reception centers [46, 47, 50], or for multigenerational households based on care and respect for weaker family members. Although implemented with the purpose of protecting the population from the risk of infection, containment measures have penalized the already disadvantaged fringes of society, thus exacerbating existing inequalities [63, 64].

We understand that vulnerability is often used as a vague and undefined concept, as pointed out by many scholars with a diverse background [69, 70]. In a recently published article, Molenaar and Van Praag objected to the vagueness of this expression used for describing the condition of migrants during the COVID-19 pandemic [38]. The authors state that the concept of vulnerability is now used as a stand-alone and ambiguous term without specifying “who is vulnerable, why they are vulnerable, and what they are vulnerable to” ([64], p.601). With this review we intended to give a concrete meaning to the concept of vulnerability with reference to migrant women in a context of disaster. While in many articles the mechanisms that produce vulnerability are absent or partially defined [38, 70], our results shed light on the causes and the paths that led migrant women to experience specific negative impacts. This can ultimately facilitate the development of strategies aimed at tackling the vulnerability of migrant women during disasters, including plans to enhance their disaster preparedness.

Certain groups are often referred to as inherently vulnerable. Their vulnerability is presented as the result of poor decisions, negative behaviors, or biological destiny [70]. Molenaar and Van Praag explain how the concept of vulnerability has been critiqued for being “patronizing and oppressive”, since it focuses on the weakness of the target group [38]. It is far from us depicting migrant women as passive and powerless subjects. Our objective was rather to provide an overview of migrant women’s experiences in order to produce recommendations for interventions aimed at improving their coping mechanisms to disasters, and also to point out the social structures that provoke the marginalization of specific groups in the society. We agree with Molenaar and Van Praag that vulnerability is not a static and given notion [38], but instead a condition subject to change. In addition, vulnerability is not only connected to the individual capacity to cope, but also to one’s own resilience, an example of which may be the ability to “access to and control over different types of resources” [2]. The negative outcomes experienced by migrant women outlined in this review should be understood in the light of the social determinants of health (SDH), namely “the conditions in which people are born, grow, work, live, and age, and the wider set of forces and systems shaping the conditions of daily life”. All these elements influence people’s health outcomes and have a meaningful impact in shaping health inequalities within society [71].

Although this review focused on the negative impacts experienced by migrant women, it is important to note that not all migrant women are equally vulnerable to the pathways described. An intersectional lens needs to be applied in order to understand the different levels of vulnerability of migrant women, taking into consideration elements such as their socioeconomic status, ability, age, or ethnicity. When we generalize the findings of this review, it must be considered that some migrant women may have had some positive experiences despite these not being portrayed in the included studies and that some women showed more resilience and strength than others. Ultimately, Molenaar and Van Praag [38] report that an indiscriminate use of the concept of vulnerability could result in further exclusion and stigmatization of groups of people, while we think that shedding light on migrant women’s vulnerability factors is paramount in order to produce and implement more inclusive policies and interventions. The best practices highlighted in the articles included in this review, such as overcoming financial or organizational difficulties through the support of others [48, 53, 56] or being helped while in need of accessing services [45, 56], offer a glimpse of how eliminating, at least partially, barriers faced by migrant women is an effective strategy that contributes to their well-being and empowerment.

### Strengths and limitations

This scoping review has a few important strengths. First, it was conducted following a systematic process, with a rigorous and transparent approach to data retrieval, screening, and analysis. Second, to the best of our knowledge, this is the first literature review exploring the pathways leading from a condition of vulnerability to negative impacts during disasters for migrant women. Third, the in-depth inductive analysis allowed us to unravel a complex multi-layered phenomenon and to be able to report the study findings in the form of pathways, which can be easily accessible to the scientific community, policymakers and the wider public. This review also has some limitations. First, not all the studies reported data systematically, and this might have affected a fuller understanding of their findings. Second, the findings of this review are limited to the COVID-19 pandemic global disaster, as studies dealing with the vulnerability of migrant women in other disasters have not been retrieved. Third, no gray literature was included in the searching process. Fourth, studies focusing on internal migrants were not included, because we decided to analyze the spectrum of barriers migrant women experience when confronted with a system that is different from the one they have in their home country. Fifth, we recognize that each host country is different from the other, also in terms of gross domestic product (GDP), and that their reception policies and integration strategies vary widely. However, in our review we decided to include studies conducted in different countries to identify common patterns in the experiences of migrant women living in a host country in the event of a disaster. Finally, migrant women are a heterogeneous group and for this reason the vulnerability factors that apply to some of them might not be relevant for others. In this regard, it was our concern to specify the type of migrant, when possible, and the study context. We also reported specific examples and quotations from original studies to facilitate the understanding of how the context can influence and shape the unique experience of each migrant woman.

### Recommendations

Literature dealing with migrant women in the context of disaster is scant. In order to strengthen disaster risk reduction (DRR) strategies, more evidence about the impact of disasters on migrant women needs to be collected. Both qualitative and quantitative research is needed, and an intersectional approach should be adopted [72]. At the same time, research should investigate coping and resilience strategies implemented by vulnerable groups, including migrant women, in order to identify examples of best practices and areas where a proper intervention is required.

We encourage authors studying vulnerable populations, including migrant women, to report disaggregated data (e.g., age, home country, legal status, lenght of stay in the host country, socioeconomic indicators) in a clear, precise, and standardized way, to allow the identification of various sub-groups collectively referred to as “migrant women”.

As pointed out by the Sendai Framework for DRR 2015–2030, there has to be a broader and a more people-centered preventive approach to disaster risk management [73]. On the same line, the H-EDRM framework called for a “whole-of-society approach” [3]. DRR should be gender-sensitive, and strategies aiming at gender equality such as gender mainstreaming [74] should be followed in line with the United Nations’ 2030 Sustainable Development Goals, in particular number five – Gender Equality [75]. Policymakers and key stakeholders should ensure that disaster risk information and health promotion strategies reach all segments of the population, paying particular attention to the most marginalized categories like migrants and refugees. Language and cultural barriers need to be overcome, primarily by relying on experts such as interpreters, cultural mediators, and anthropologists.

Health equity is realized when everyone can attain their full potential for health and well-being [76]. The COVID-19 pandemic demonstrated that the achievement of this goal is still far to reach [77]. The post-COVID-19 era should build upon the lessons learned during the pandemic and constitutes the momentum for thoroughly identifying and addressing the SDH that perpetuate inequalities and penalize the most vulnerable and marginalized groups [78].

## Conclusion

This scoping review provided an analysis of the vulnerability of migrant women and the pathways leading to negative outcomes during a disaster. In the reviewed literature, legal status and poverty conditions were the vulnerability factors linked with most of the reported negative impacts. On the other side, the worsening of their mental health status was the outcome influenced by all the identified vulnerability factors. This review contributed to give a concrete meaning to the concept of migrant women’s vulnerability and it shed light on the importance of addressing the Social Determinants of Health when developing inclusive disaster preparedness interventions. Scientific literature on this topic proved to be scant. Both qualitative and quantitative research is required.

## Supplementary Information

**Additional file 1.** Search strings per each database. Search strings used to retrieve articles from PubMed, Scopus and Web of Science.

**Additional file 2.** Extraction sheet. Extraction sheet used to collect data from retrieved articles.

**Additional file 3.** Vulnerability pathways for migrant women during COVID-19. Framework depicting the vulnerability pathways experienced my migrant women during the COVID-19 pandemic.

## Data Availability

The datasets used and/or analysed during the current study are available from the corresponding author on reasonable request.

## Abbreviations

H-EDRM: Health Emergency and Disaster Risk Management
WHO: World Health Organization
COVID-19: Coronavirus Disease 2019
GBV: Gender-Based Violence
PRISMA-ScR: Preferred Reporting Items for Systematic reviews and Meta-Analyses extension for Scoping Reviews
HIV: Human Immunodeficiency Virus
FGD: Focus Group Discussion
PRISMA: Preferred Reporting Items for Systematic reviews and Meta-Analyses
USA: United States of America
HCA: Health Care Aid
LTC: Long-Term Care
HCW: Health Care Worker
UK: United Kingdom
SGBV: Sexual and Gender-Based Violence
IPV: Intimate Partner Violence
NGO: Non-Governmental Organization
PPE: Personal Protective Equipment
SDH: Social Determinants of Health
GDP: Gross Domestic Product
DRR: Disaster Risk Reduction
