# Supplementary material for: Vulnerability of migrant women during disasters: a scoping review of the literature
Source: Int J Equity Health. 2023 Jul 22;22:135. doi: 10.1186/s12939-023-01951-1 (PMC10362632; doi:10.1186/s12939-023-01951-1)
Supplement: Supplementary file 1 — Additional file 1. Search strings per each database. Search strings used to retrieve articles from PubMed, Scopus and Web of Science. [file 12939_2023_1951_MOESM1_ESM.docx]

**Additional file 1**

**Table 1** Search strings per each database.

| **Database** | **Retrieved records** | **Search string** |
| --- | --- | --- |
| PubMed | 737 | ("transients and migrants"[MeSH Terms] OR "migrant"[Title/Abstract] OR "refugee*"[Title/Abstract] OR "migrat*"[Title/Abstract] OR "asylum seeker*"[Title/Abstract] OR "expat*"[Title/Abstract]) AND ("women"[Title/Abstract] OR "woman"[Title/Abstract] OR "female*"[Title/Abstract] OR "gender"[Title/Abstract] OR "girl*"[Title/Abstract]) AND ("disaster*"[MeSH Terms] OR "disaster*"[Title/Abstract] OR "covid*"[Title/Abstract] OR "earthquake*"[Title/Abstract] OR "flood*"[Title/Abstract] OR "heat wave*"[Title/Abstract] OR "heatwave*"[Title/Abstract] OR "storm*"[Title/Abstract] OR "pandemic*"[Title/Abstract] OR "epidemic*"[Title/Abstract]) |
| Scopus | 3122 | (TITLE-ABS-KEY ( migrant* OR refugee* OR migrat* OR "asylum seeker*" OR expat* ) AND TITLE-ABS-KEY ( women OR woman OR female* OR gender OR girl* ) AND TITLE-ABS-KEY ( disaster* OR covid* OR earthquake* OR flood* OR "heat wave*" OR heatwave* OR storm* OR pandemic* OR epidemic*)) |
| Web of Science | 1163 | TOPIC: migrant* OR refugee* OR migrat* OR "asylum seeker*" OR expat* AND TOPIC: women OR woman OR female* OR gender OR girl* AND TOPIC: disaster* OR covid* OR earthquake* OR flood* OR "heat wave*" OR heatwave* OR storm* OR pandemic* OR epidemic* |
