# Supplementary material for: Vulnerability of migrant women during disasters: a scoping review of the literature
Source: Int J Equity Health. 2023 Jul 22;22:135. doi: 10.1186/s12939-023-01951-1 (PMC10362632; doi:10.1186/s12939-023-01951-1)
Supplement: Supplementary file 2 — Additional file 2. Extraction sheet. Extraction sheet used to collect data from retrieved articles. [file 12939_2023_1951_MOESM2_ESM.docx]

**Table 2** Extraction sheet.

| General information of the article | Title |
| --- | --- |
|  | Authors |
|  | Publication year |
|  | Language |
|  | Country where the study was conducted |
| Study design | Study period |
|  | Objective of the study |
|  | Study type |
|  | Methodology |
|  | Population / Source of data |
|  | Population of comparison (if any) |
| Information about the migrant experience | Type of migrant |
|  | Age of migrants |
|  | Home country |
|  | Host country |
|  | Lenght of migrant status |
| Vulnerability | Type of disaster |
|  | Type of hazard |
|  | Event |
|  | Factors that determine vulnerability |
|  | Negative outcome |
|  | Layers of impact |
| Limitations | Study limitations (authors) |
|  | Study limitations (us) |
| Notes | Notes |
| References | Articles retrieved from references |
