# Supplementary material for: Vulnerability of migrant women during disasters: a scoping review of the literature
Source: Int J Equity Health. 2023 Jul 22;22:135. doi: 10.1186/s12939-023-01951-1 (PMC10362632; doi:10.1186/s12939-023-01951-1)
Supplement: Supplementary file 3 — Additional file 3. Vulnerability pathways for migrant women during COVID-19. Framework depicting the vulnerability pathways experienced my migrant women during the COVID-19 pandemic. [file 12939_2023_1951_MOESM3_ESM.pptx]

## Slide 1
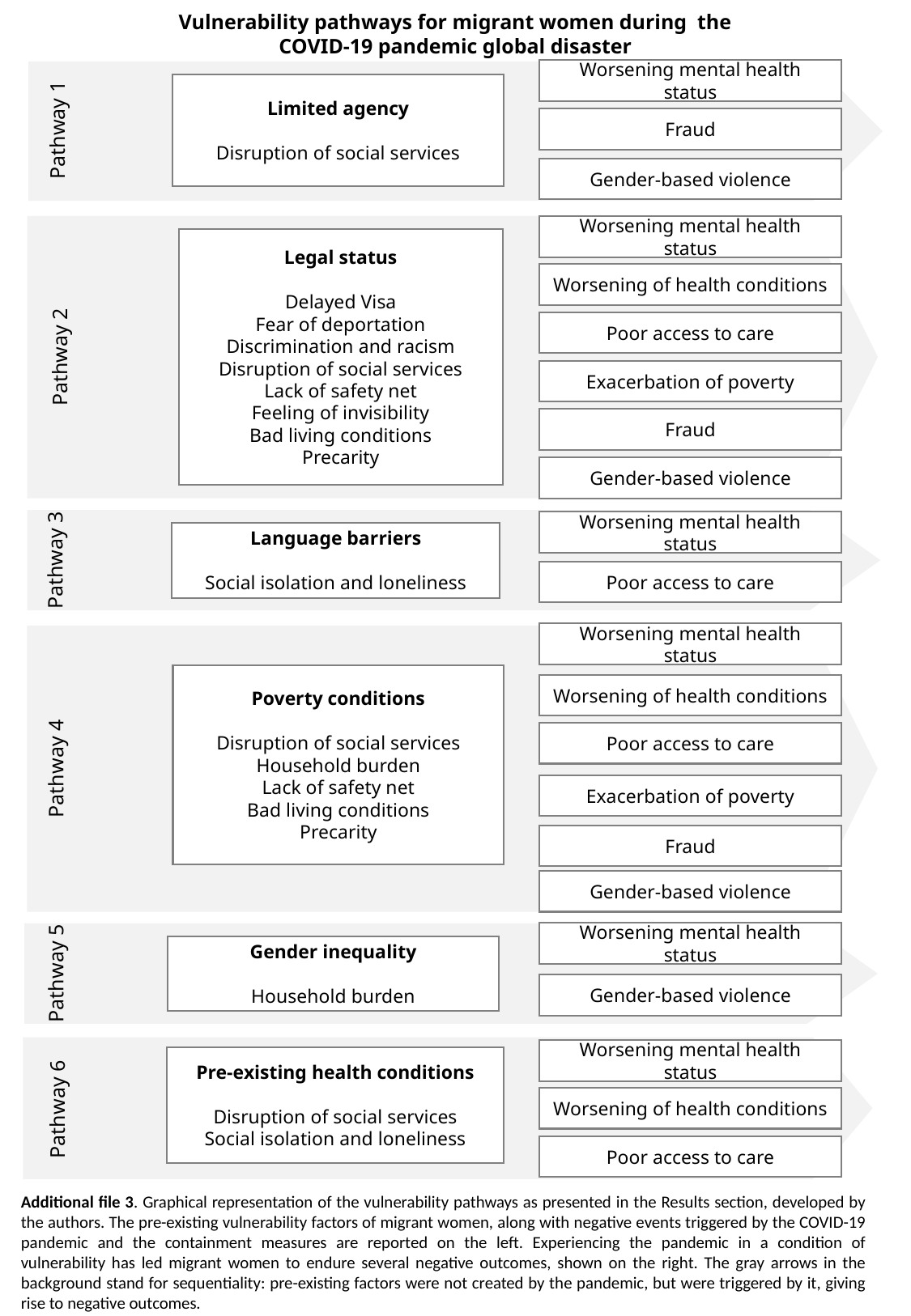

Vulnerability pathways for migrant women during the COVID-19 pandemic global disaster
Worsening mental health status
Limited agency
Disruption of social services
Fraud
Pathway 1
Gender-based violence
Worsening mental health status
Legal status
Delayed Visa
Fear of deportation
Discrimination and racism
Disruption of social services
Lack of safety net
Feeling of invisibility
Bad living conditions
Precarity
Worsening of health conditions
Poor access to care
Pathway 2
Exacerbation of poverty
Fraud
Gender-based violence
Worsening mental health status
Language barriers
Social isolation and loneliness
Pathway 3
Poor access to care
Worsening mental health status
Poverty conditions
Disruption of social services
Household burden
Lack of safety net
Bad living conditions
Precarity
Worsening of health conditions
Poor access to care
Pathway 4
Exacerbation of poverty
Fraud
Gender-based violence
Worsening mental health status
Gender inequality
Household burden
Pathway 5
Gender-based violence
Worsening mental health status
Pre-existing health conditions
Disruption of social services
Social isolation and loneliness
Worsening of health conditions
Pathway 6
Poor access to care
Additional file 3. Graphical representation of the vulnerability pathways as presented in the Results section, developed by the authors. The pre-existing vulnerability factors of migrant women, along with negative events triggered by the COVID-19 pandemic and the containment measures are reported on the left. Experiencing the pandemic in a condition of vulnerability has led migrant women to endure several negative outcomes, shown on the right. The gray arrows in the background stand for sequentiality: pre-existing factors were not created by the pandemic, but were triggered by it, giving rise to negative outcomes.
